# Applying the perturbative integral in aeromaneuvers around Mars to calculate the cost

**DOI:** 10.1038/s41598-022-08830-9

**Published:** 2022-03-23

**Authors:** Jhonathan O. Murcia Piñeros, Antônio F. Bertachini de Almeida Prado, Walter Abrahão dos Santos, Rodolpho Vilhena de Moraes

**Affiliations:** 1 Institute of Science and Technology ICT-UNIFESP, São José dos Campos, SP Brazil; 2 Graduate Division - DIPGR, National Institute for Space Research INPE, São José dos Campos, SP Brazil; 3 Small Satellite Division - DIPST, National Institute for Space Research INPE, São José dos Campos, SP Brazil; 4 Peoples’ Friendship University of Russia (RUDN University), 6 Miklukho-Maklaya, Moscow, 117198 Russian Federation

**Keywords:** Astronomy and planetary science, Engineering, Aerospace engineering

## Abstract

The perturbative integral method was applied to quantify the contribution of external forces during a specific interval of time in trajectories of spacecraft around asteroids and under the Luni-solar influence. However, this method has not been used to quantify the contributions of drag in aerocapture and aerobraking. For this reason, the planet Mars is selected to apply this method during an aerogravity-assisted maneuver. Several trajectories are analyzed, making use of a drag device with area to mass ratios varying from 0.0 to 20.0 m^2^/kg, simulating solar sails or de-orbit devices. The mathematical model is based in the restricted three-body problem. The use of this maneuver makes it possible to obtain the variations of energy in the trajectory, replacing expensive maneuvers based on fuel consumption. To observe the effects of the maneuvers, different values of pericenter velocity and altitude were selected for prograde and retrograde orbits. The innovation of this research is the application of an integral method to quantify the delta-V of the aero gravity maneuver, comparing the cost of the maneuver with the traditional methods of space propulsion. The results allow the identification of orbits with conditions to capture, and the perturbative maps show the velocity variations.

## Introduction

A method to quantify the influence of orbital perturbations called Perturbative Integral (PI) was presented in Refs.^1–3^. The PI quantifies the contribution of each acceleration involved in the spacecraft trajectory. It was implemented to find the less perturbed orbits around the Earth and under Lunisolar influence^2^. It was also applied to map orbits around asteroids^3^ and to analyze the effect of the terms of the expansion of the gravity field of the Earth^1^. Other applications include the quantification of perturbative forces in harmonic and duffing oscillators and measurements of the perturbations in satellites^4^. Those ideas were later generalized, and four types of PI were described in the literature to calculate perturbation maps around asteroids^5^ and to quantify the effects of the accelerations during a Powered Aero-Gravity Assisted Maneuver (PAGAM)^6^. The results showed that the PI measures the evolution of the perturbations, indicating the Delta-V (DV) given by those forces and so predicting the orbital changes they made in the trajectory of the spacecraft. An important improvement appeared in the literature when studying the irregular shape of the Earth^7^, which made an important step in this type of integral index and developed a version that is much more accurate in several types of applications when there are forces in opposed directions involved in the trajectory.

As was presented, the PI was applied in several problems in orbital dynamics, however, it has not been used to analyze aeromaneuvers, with a focus on aerocapture, which is useful for future interplanetary flights. For example, the PI could be applied to quantify the contribution of the aerodynamic forces during a final approach in planets like Venus, Mars, and Jupiter, which present a significant atmospheric density. Conceptual missions are projected to include aerocapture and aerobraking maneuvers, instead of traditional propulsion systems, reducing the costs of the mission^8–12^.

Successful missions to Mars, like the InSight (2018), showed that the interest in the exploration of the planet continues to grow. A highlight of InSight was the transportation of two small spacecraft: MarCO A and MarCO B, the first interplanetary CubeSats. The sizes of the SmallSats restricted the use of traditional propulsion systems, due to the limited mass and volume. This scenario allows the exploration of alternative techniques as aeromaneuvers, which are possible due to the implementation of drag devices, a low-cost technology with a higher level of maturity, compared to the small propulsion systems for NanoSats.

Considering the use of small spacecraft in space exploration missions, the maturity of the technology, and the future missions to Mars, the present paper contributes to this topic by applying the PI technique to calculate the contribution of the drag during aeromaneuvers. The idea is to measure the variation of velocity given by drag during the whole trajectory of the spacecraft, so the savings in propulsion systems can be measured and expressed by the PI scalar index.

Due to the close approach, the resulting trajectory could be a Gravity Assisted Maneuver (GAM), or swing-by, and, with the pericenter located inside the atmosphere, in an Aero-Gravity Assisted Maneuver (AGAM)^13–16^. If the conditions are optimal, the approach can end in aerocapture. Different studies about the aerocapture have been discussed in the scientific literature^8–12,17–20^, showing the DV savings due to the aerodynamic effect of the atmosphere of Mars.

The next section describes the mathematical model of the trajectory and the mapping techniques of the PI used to quantify the contribution of drag as a function of the spacecraft’s Area-to-Mass ratio (*A/m*), the methodology, followed by a discussion of the results obtained and the conclusions.

## Modeling the trajectory of the spacecraft

The mathematical model selected for the simulations is the Circular Restricted Three-Body Problem (CRTBP), a model that was implemented with success in the analysis of GAM and AGAM^6,13–15^. The orbit of the spacecraft is studied around the center of mass of the system Sun-Mars. The Sun and Mars are assumed to be moving in circular Keplerian orbits around their common center of mass, and the spacecraft (third body with infinitesimal mass) is approaching Mars. In this case, the spacecraft has a drag device to increase the *A/m*, generating a larger effect of aerodynamic deceleration (*AD*). At the beginning of the simulations, the spacecraft is orbiting the Sun in a transfer orbit from the Earth, and then it makes a passage inside the atmosphere of Mars.

### The AGAM

The dynamic equations in the plane of the rotating coordinate system in dimensionless variables, and restricted to the plane of the primaries, are derived from the potential of the CRTBP^13^ with the addition of the acceleration due to drag, in a 2D maneuver. The two dynamical equations, as a function of the potential $\Omega$, are:

1 $$\ddot{x}=2\dot{y}+{\Omega }_{x}+ADx$$

2 $$\ddot{y}=-2\dot{x}+{\Omega }_{y}+ADy$$

The potential is a function of the spacecraft´s position vectors to the Sun $\left({r}_{1}=\sqrt{{\left(x+\mu \right)}^{2}+{y}^{2}}\right)$, to Mars $\left({r}_{2}=\sqrt{{\left(x-1+\mu \right)}^{2}+{y}^{2}}\right)$ and to the gravitational constant relative to the mass of Mars *(μ*). The potential function is:

3 $$\Omega =\frac{1}{2}\left({x}^{2}+{y}^{2}\right)+\frac{\left(1-\mu \right)}{{r}_{1}}+\frac{\mu }{{r}_{2}}$$

The derivation of the Eqs. (1) to (3) is detailed in Ref.^13^. Using the variables of the system, the general form of the orbital energy of the spacecraft with respect to Mars is:

4 $$\upvarepsilon =\frac{{{V}_{2}}^{2}}{2}-\frac{\mu }{{r}_{2}}$$

### Drag

The drag is a dissipative force acting in the direction opposite to the motion of the spacecraft relative to the atmosphere (*V*_*2*_). The acceleration given by drag is a function of the *A/m*, the atmospheric density ($\rho$), and the drag coefficient (*C*_*D*_), which is assumed to be constant (2.0 for the present scenario, which represents a flat plate in rarefied flow). The acceleration due to drag is described in more detail in^21^, and it is represented by:

5 $$\overrightarrow{AD}=-\frac{{C}_{D}}{2}\left(\frac{A}{m}\right)\rho {V}_{2}\overrightarrow{{V}_{2}}$$

For practical considerations, the constant value along the trajectory (*C*_*D*_*A/m*) is known as the ballistic parameter (*B*).

Equation (6) shows the exponential model to calculate the density as a function of the altitude $h$, where ${\rho }_{0}$ is the atmospheric density at the surface of Mars (0.020 kg/m^3^) and *H* is the scale height (11,000 m)^22^. The upper limit of the atmospheric model is assumed to be 2500 km of altitude.

6 $$\rho ={\rho }_{0}{\mathcal{e}}^{-\frac{h}{H}}$$

### Perturbative integral

This method measures the variation of the velocity of the spacecraft due to the drag during the whole period of the atmospheric passage. The *PI* is defined as the integral of the *AD*, from the beginning of the atmospheric passage (*t*_*i*_) to the end of the passage inside the planetary atmosphere (*t*_*f*_). In this case, it is selected the version of the *PI* that integrates the magnitude of the acceleration, because there is no compensation of accelerations coming from different directions during the trajectory, since the drag always removes energy from the spacecraft^5^. The study is made numerically, so the assumption of Keplerian orbits is not made. Equation (7) defines the *PI* used here.

7 $$PI=\underset{{t}_{i}}{\overset{{t}_{f}}{\int }}\left|\overrightarrow{AD}\right|dt$$

## Parameters of the simulations

Prograde and retrograde trajectories are used in the simulations made in the present paper. The pericenter altitude is selected to be inside the region where the atmosphere is dense enough to generate measurable perturbative effects, and the velocity at this point is calculated as the excess of velocity from a transfer orbit, to guarantee the approaching in hyperbolic conditions. Since the focus of the present research is on the implementation of the PI during the aeromaneuvers, the mathematical model is ideal, without uncertainties and/or additional perturbations. A more detailed analysis, including a dynamical model of the atmosphere and the variability of the density could be part of future research.

To determine the initial conditions of the AGAM from the selected pericenter, the trajectories are propagated in negative times using the GAM, stopping when a distance of 0.5 DU from the center of Mars is reached. During this propagation, it is monitored the conservation of the Jacobi Constant with an accuracy lower than 1 × 10^–14^^6,14,15^. In the case of the AGAM, it is selected an A/m interval from 0.0 m^2^/kg to 20.0 m^2^/kg (the minimum value is used for the GAM and the maximum value represents the presence of solar sails or high drag devices). After one day of the passage of the spacecraft by the pericenter, the two-body energy relative to Mars and its distance is evaluated to classify the resulting trajectory, because the value of the energy indicates which type of trajectory the spacecraft has relative to Mars after the first approach.

The mathematical model was coded using the numerical integrator RKF-7/8 to solve the dynamic equations of motion (1) and (2). In the case of Eq. (7), it was integrated numerically, applying Simpson´s rule.

Due to the use of the dimensionless variables, the equivalent values of the non-dimensional units of the systems in the SI are presented in Table 1.

**Table 1:** Equivalence of the Non-dimensional Units Sun-Mars to SI.

Canonical Unit	Equivalence in SI
Distance Unit (1 DU)	1.52367934 AU (2.27939186 × 10^11^ m)^21^
Velocity Unit (1 VU)	24,131.229 m/s
Time Unit (1 TU)	1 rad (2,623.8381442 h)
Period of primaries	2 rad$\pi$ (16,486.0613 h)
Mars Radius (1 MR)	3,397 200 m^21^
$\mu =\frac{{m}_{Mars}}{\left({m}_{Sun}+{m}_{Mars}\right)}$	3.227136860 × 10^–7^

## Mapping the resulting orbits

The trajectories were propagated with *A/m* increasing in steps of 0.04 m^2^/kg to cover the effect of the deployment of the drag device. Initial perigee altitudes (h_p_) change from 100 to 150 km, in steps of 0.1 km.

For the trajectories arriving from the transfer orbit in GAM, two velocities at the pericenter of Mars were selected, 0.246 and 0.336 VU, which are calculated from a Hohmann transfer assuming a perihelium equal to the Earth´s semimajor-axis and an aphelion larger than the semimajor-axis of Mars, to generate a hyperbolic trajectory. The initial points are the same for all the trajectories (GAM and AGAM), since the trajectories come from the Earth. The angle of approach was selected to be 0°, indicating that the pericenter is in the apsis line, in front of the planet and in the line of the apsides.

**Figure 1:**
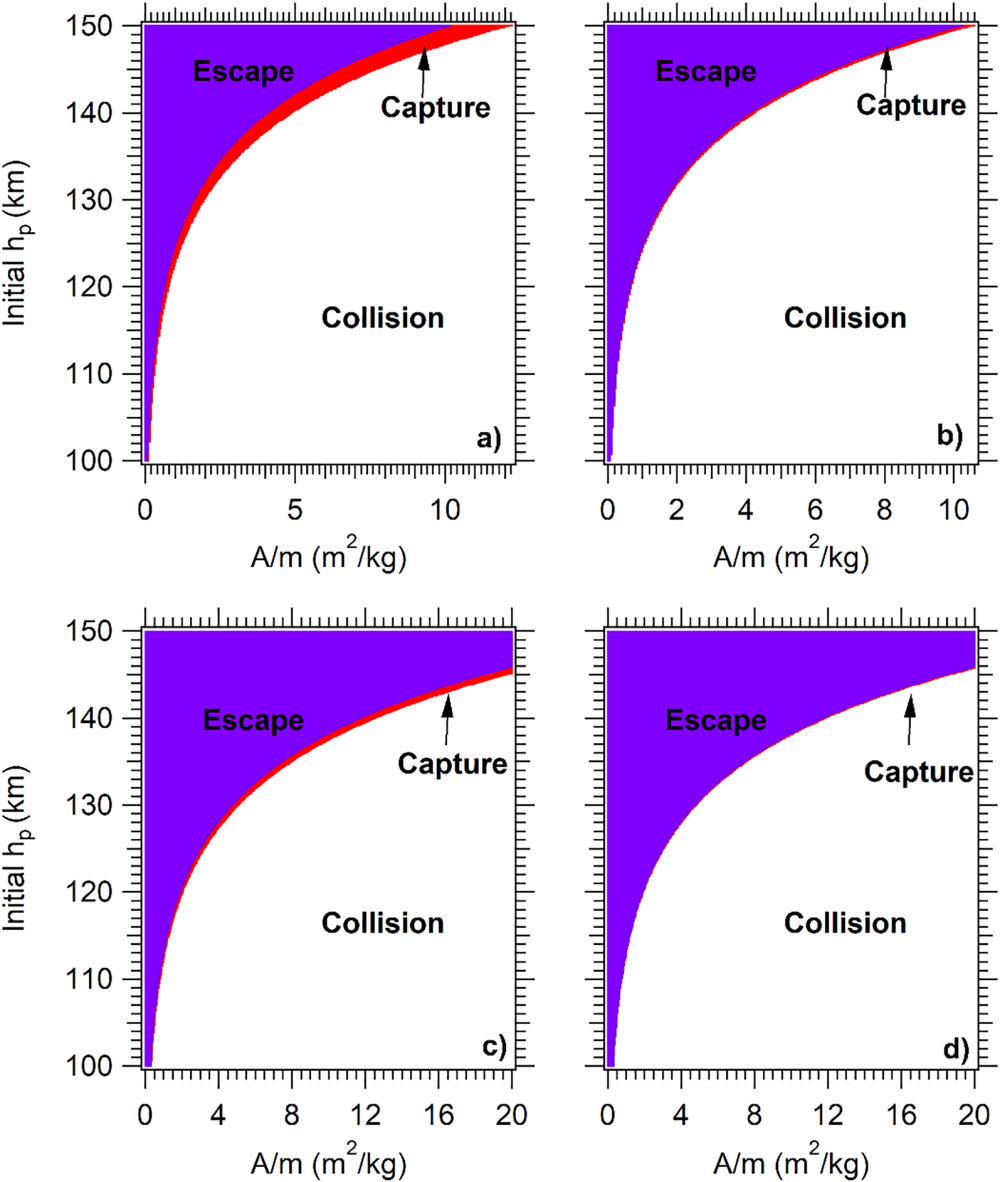
Final trajectories for: (**a**) V_P_ = 0.246 VU and t = 1 day, (**b**) V_P_ = 0.246 VU and t = 30 days, (**c**) V_P_ = 0.336 VU and t = 1 day, (**d**) V_P_ = 0.336 VU and t = 30 days. In this case, the time are days after the passage.

Figure 1 shows the three resulting trajectories, classified in color scale, according to the value of the energy after the passage and in prograde direction. White color represents trajectories that, after the approach, ended in collisions with the planet. This region is increased for larger values of *A/m* and does not appear for the GAM (*B* = 0.0 m^2^/kg). Just above the collision region, the capture zone (red color) appears. After the capture, the continuum interaction of the spacecraft with the atmosphere generates aerobraking trajectories. It is possible to observe the aerobraking effect in the changes in the red regions of Fig. 1. The capture region is the smallest of the three regions, because it depends on the specific combination between *A/m*, atmospheric density, and velocity, being sensitive to small density variations (in this case as a function of altitude). The losses of energy in this region have intermediary values, which are large enough to transform the hyperbolic trajectory into an elliptic orbit (capture), but not large enough to make a reentry in less than one day.

The last region of Fig. 1 is represented in blue color. The trajectories located in this region are hyperbolic trajectories before and after the passage (without capture), which means that a successful AGAM occurred, and the combination of the gravity and the atmosphere of Mars were used to modify the interplanetary trajectory of the spacecraft, reducing the energy due to drag.

Figure 1a,b show the resulting trajectories after the passage of the spacecraft by the pericenter with a velocity of 0.246 VU. All the trajectories with *A/m* larger than 12 m^2^/kg ended in collisions less than one day after the close approach (Fig. 1a). After 30 days of propagation, maintaining A/m constant, without external perturbations and without additional controls, the white region increases, and trajectories with A/m larger than 10.8 m^2^/kg also ended in collisions due to the continuum braking (Fig. 1b). This continuum breaking reduces the red region by 30 days after the capture (Differences between Fig. 1a,b).

For 0.336 VU, the white and red regions are reduced, and the number of escapes increase**s**, which indicates that the increase of the velocity generates higher values of drag, but also reduces the duration of the passage, reducing the effect of the perturbation so making captures more difficult to occur (Fig. 1c,d). The differences observed between the plots above and below in Fig. 1 show the decrease in the escape altitude region and the reduction in the number of captured trajectories, due to the increase in the velocity of the passage. The scenarios for 0.336 VU and altitudes larger than 140 km are ideal for the AGAM when this maneuver is desired, like in the case of the returning of a spacecraft to the Earth, or to send it to other parts of the solar system (see Fig. 1c,d).

The aerobraking maneuver is useful to place the spacecraft in a final or in a parking orbit, keeping it in orbit for longer times with the reduction of A/m. In other words, the drag device is deployed for the aerocapture and removed when it is desired to reduce the effects of the atmospheric drag (aerobraking), searching to stabilize the orbit of the spacecraft for a long period. In this case it is possible the use active control to keep the orbit.

A total of 250,000 trajectories were simulated for each scenario. For approaches with a velocity of 0.246 VU, one day after the passage by the pericenter, only 5,536 trajectories resulted in aerocapture. After one month, this number was reduced to 943, due to the aerobraking, which reduced the limit of the altitude ending in collision (Fig. 1a,b). For the scenario with 0.336 VU, 3,673 trajectories were captured after the passage, and one month later only 620 trajectories survived (red region in Fig. 1c,d). It means that the complete mission must plan maneuvers to stabilize the orbit after insertion, but 30 days is a time long enough to do those maneuvers with all safety measures that are required. Therefore, the aerobraking is an intermediate phase that reduces the energy of the trajectory of the spacecraft, reducing the fuel consumption to complete the maneuver.

**Figure 2:**
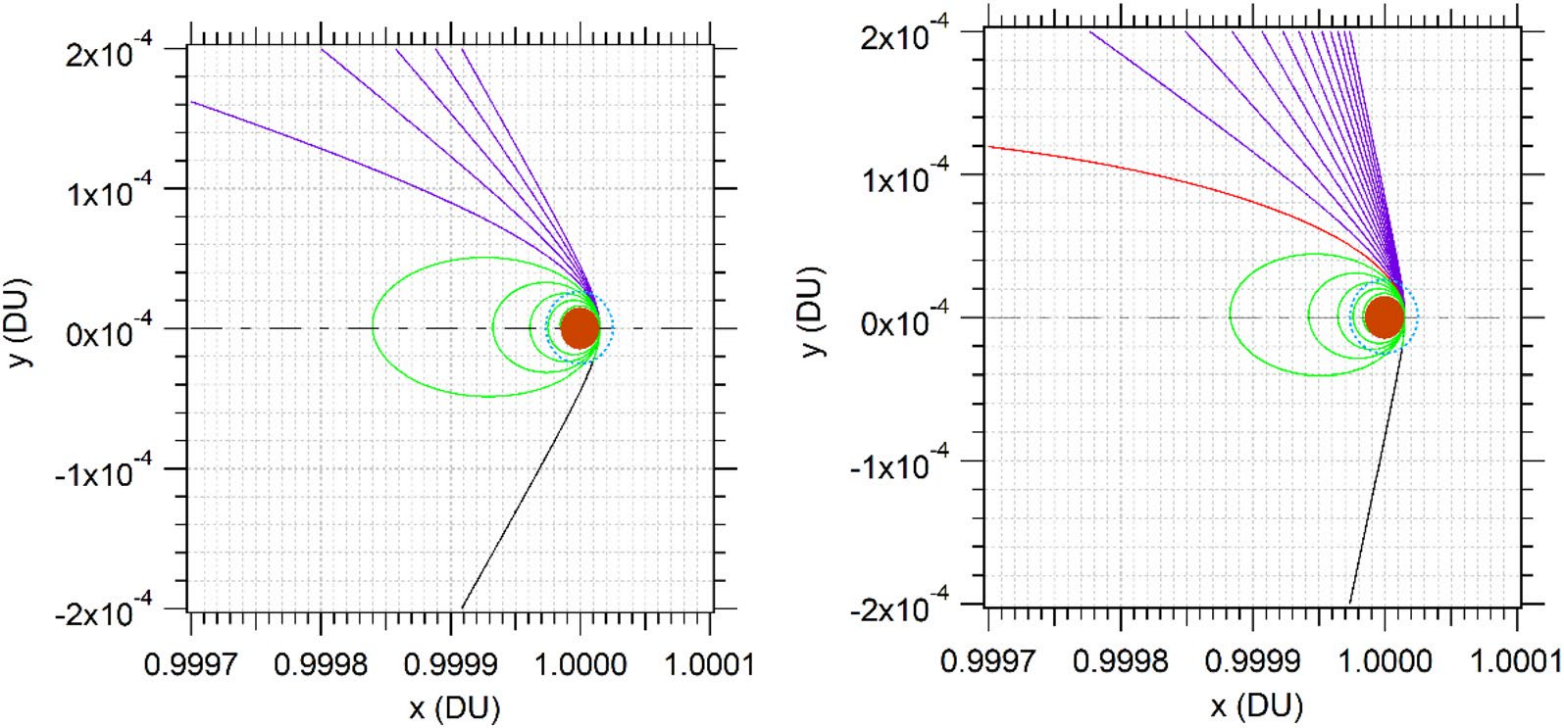
Trajectories in the synodic frame with V_P_ = 0.246 VU (left) and 0.336 VU (right).

The resulting orbits after one day of propagation are presented in Fig. 2. In this case, it was selected an interval of A/m of 1.0 m^2^/kg, at 140 km of h_p_, after 1 day of the passage. Initial GA trajectories are represented in black (zero drag), escape in blue, captures in red, and captures ending in collisions in green. Mars is the orange circle and the atmospheric limits are shown by the blue dots. It is possible to observe the resulting geometry of the orbit after the closest approach. The trajectories are plotted in the plane of the synodic frame (see Fig. 2). With the increase of velocity (Fig. 2. right), it is possible to observe the increase in AGAM or escape trajectories, with a lower curvature angle.

**Figure 3:**
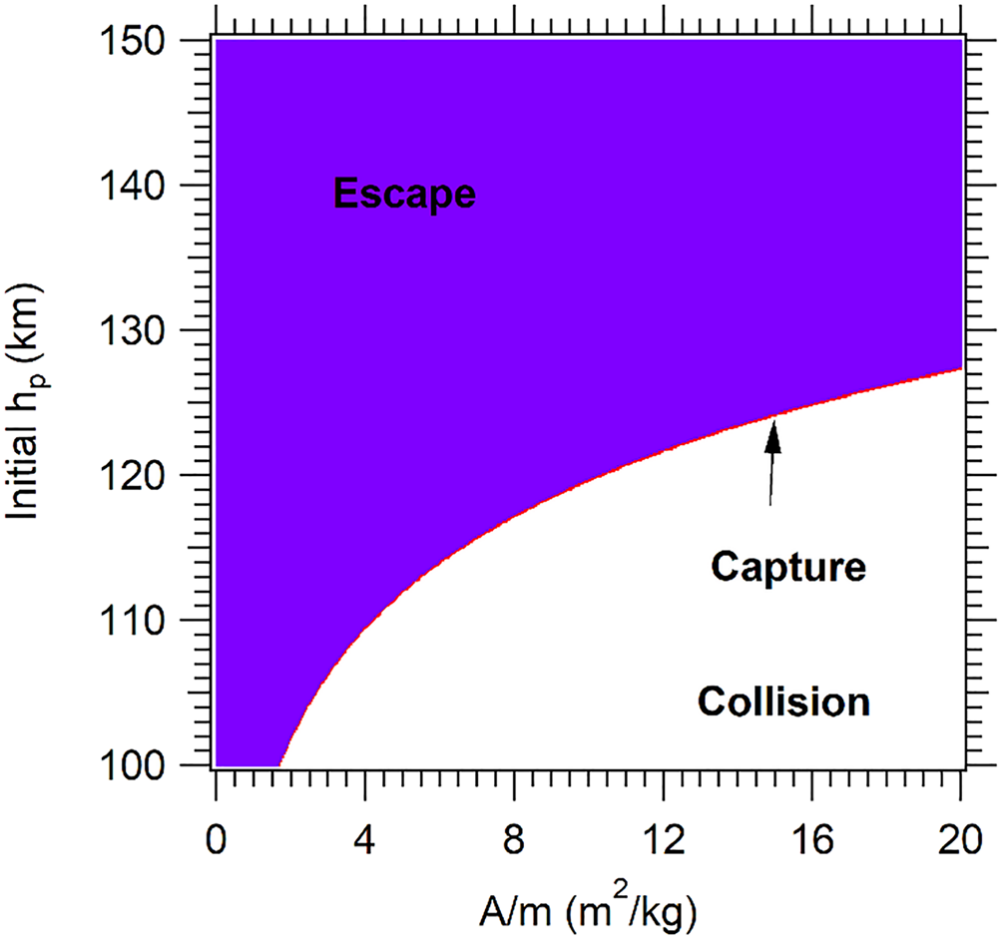
Final trajectories for V_P_ = 1.89 VU, retrograde direction.

The second case analyzed here (or the third scenario) is the retrograde motion, which main effect is the increase in the relative velocity between the spacecraft and the atmosphere. In the rotational frame, the resulting V_P_ is 1.89 VU, then, in this case, the velocity relative to the atmosphere is maximized, reducing the collision regions, and increasing the number of escape trajectories, compared to the other scenarios. It was observed that only 796 orbits resulted in captures after one day of the approach, as shown in Fig. 3. In this case, for 30 days after the passage, all the captured trajectories continued in aerobraking, ending in collisions, and then the red region turns white. Due to the velocity, the effect of aerobraking is larger than in the prograde trajectories so reducing the time of the captured orbits.

From Figs. 1, 2, 3, it is possible to conclude that the quantity of captures is maxima when the periapsis reference velocity for the GA is lower (in this case 0.246 VU, see Fig. 1a,b). With the increase of the velocity at the periapsis of Mars, the capture region is reduced, both in the number of captures and in the altitude of the region, so moving the graphics to the right inferior corner, obtaining an increase in the escapes, and reducing the number of collisions. It is possible to observe that one of the advantages of larger velocities at the aphelion is the reduction of direct collisions, but it also reduces the number of captures (aerocapture red region).

**Figure 4:**
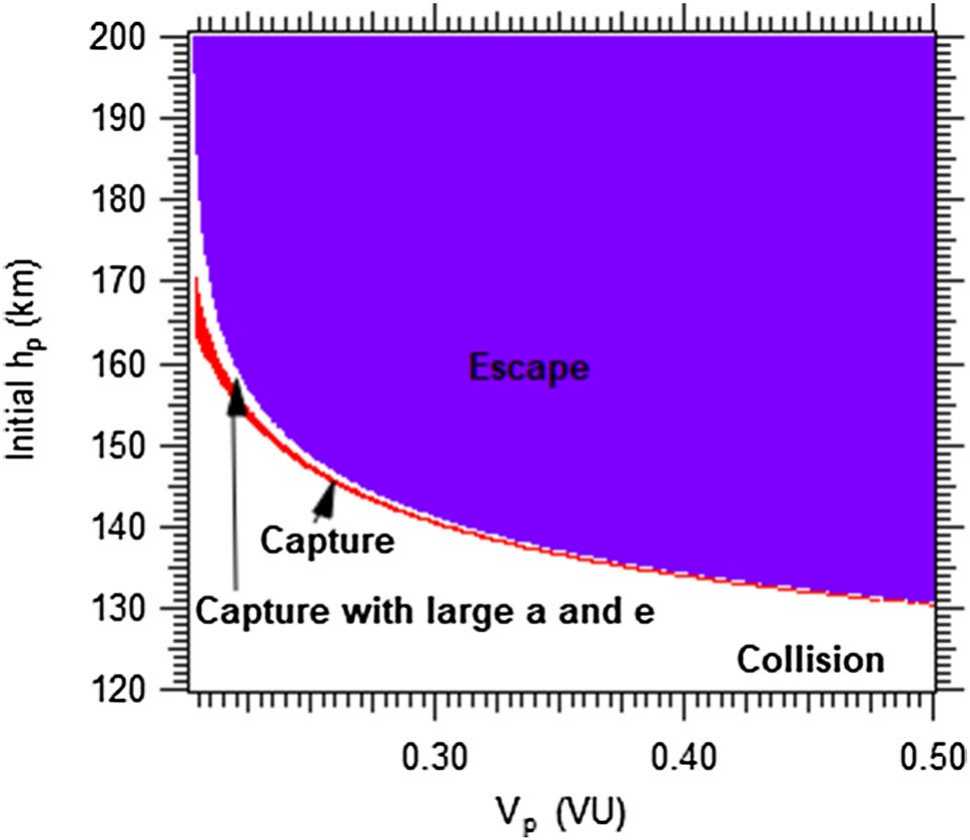
Final trajectories for A/m = 10.0 m^2^/kg, t = 1 day after the passage.

Figure 4 shows the limits of the capture region as a function of the altitude and the reference velocity at the pericenter of Mars, as calculated from an ideal GA in a prograde direction. In Fig. 4 it is observed that the increase of velocity reduces the collision region at the lowest altitudes, so increasing the escape region. The white region, between the red and blue regions, represents the capture trajectories ending in high eccentricity and large value of the semi-major axis, after 1 day of the passage, because they are closer to the escape region with largest eccentricity. Then, these trajectories will collide with the planet in the next passage by the pericenter.

The variations of the angle of approach for a selected altitude and velocity at periapsis during a GA, generate changes in the energy and velocity after the close approach^15^. In this case, it was analyzed the influence of the angle of approach in the collision, capture, and escape regions. The results show that the regions are constant for a selected velocity of approach and *A/m*, independent of the angle of approach, since the relative velocity to the atmosphere is the same.

## Mapping the resulting orbits

The resulting trajectories after the closest approach (aerocapture and AGAM), are analyzed using the PI technique, which means that we will measure the value of the total variation of velocity (DV) given by the drag. This method can measure the reduction in costs of fuel obtained by the AGAM because the PI measures the contribution of the atmosphere to the maneuver, which is equivalent to the impulse necessary to reduce the velocity of the spacecraft to place it in its final orbit.

**Figure 5:**
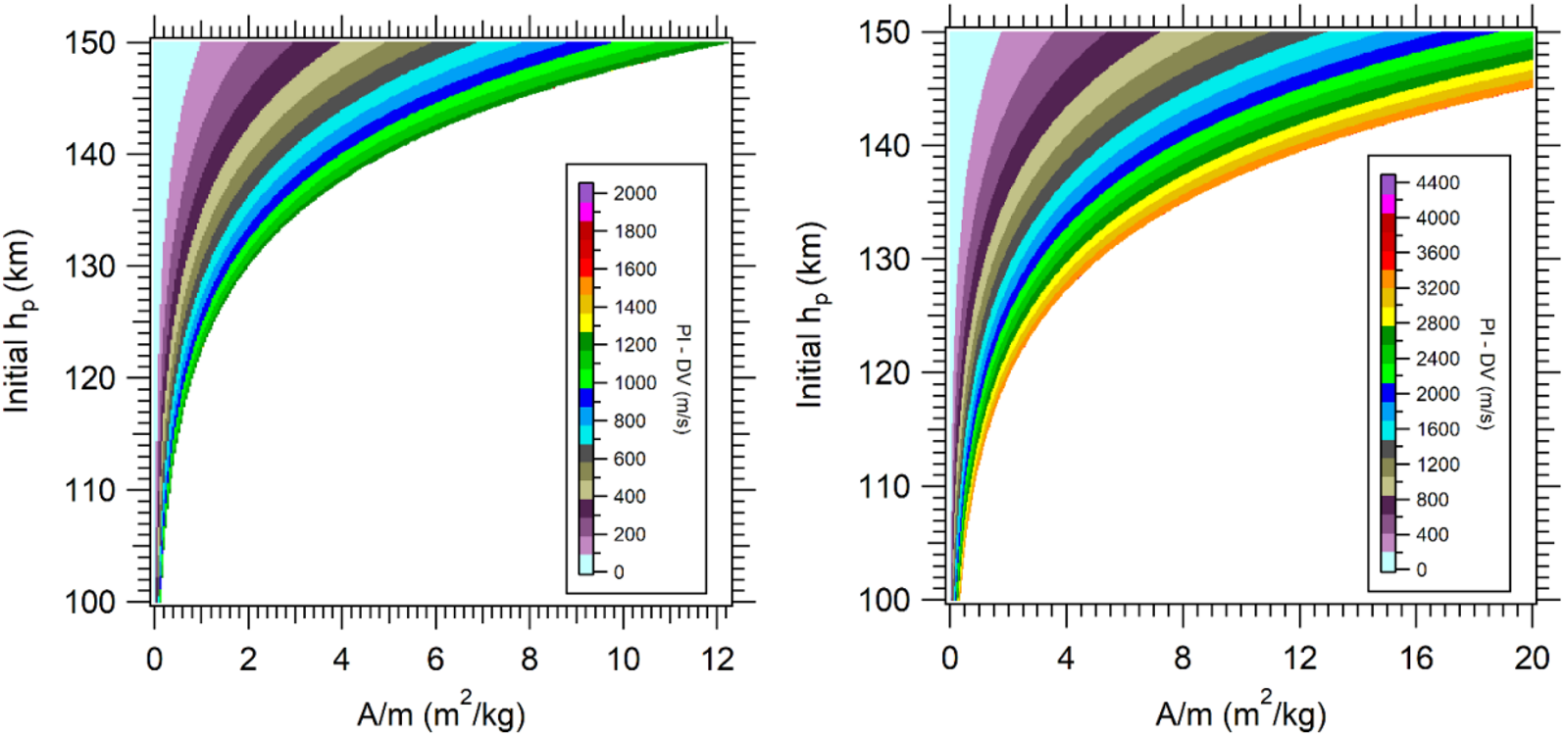
PI maps for V_P_ = 0.246 VU (left), 0.336 VU (right).

**Figure 6:**
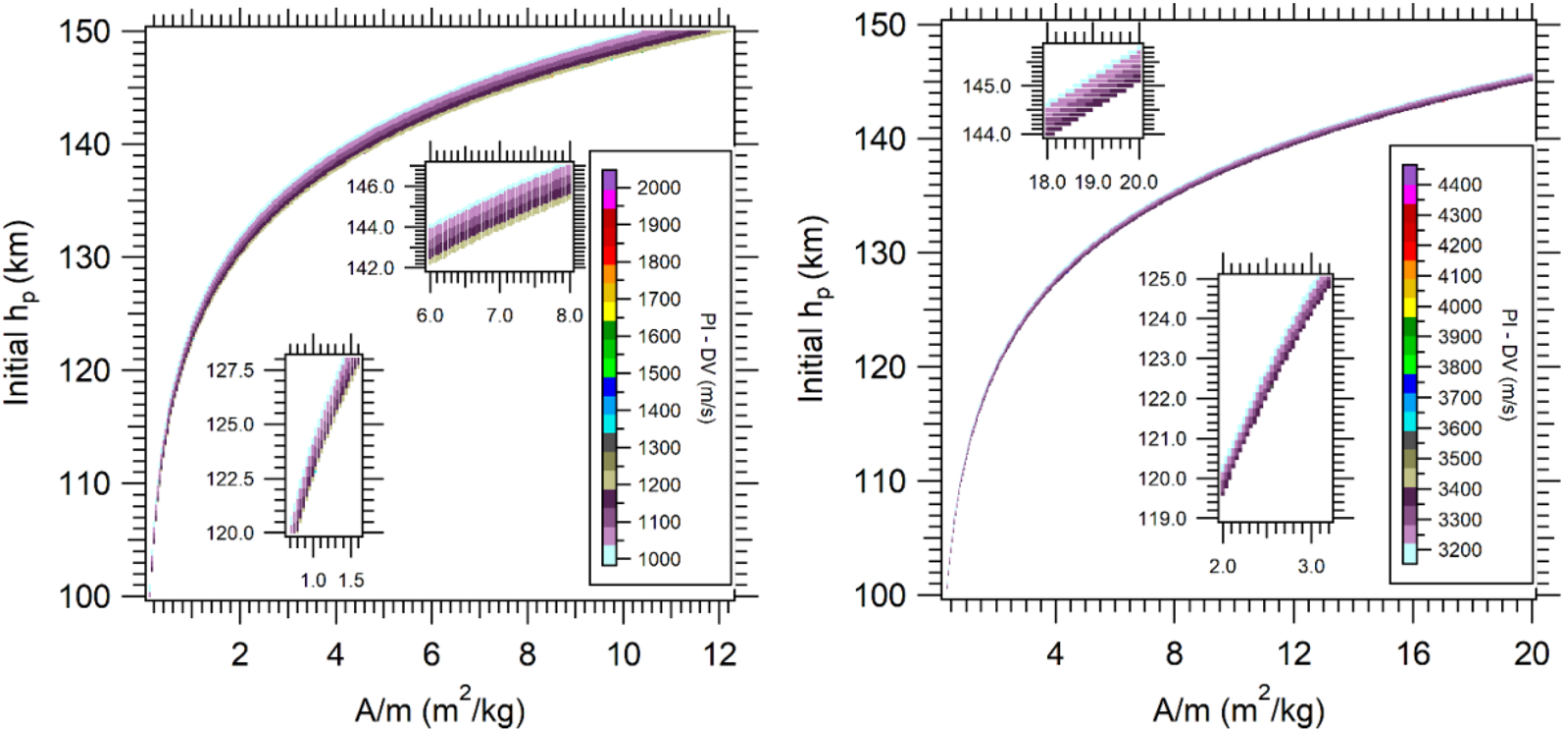
PI maps for aerocapture, V_P_ = 0.246 VU (left), 0.336 VU (right).

With the use of the PI maps, it is possible to find the existence of a lower limit of 1.0 × 10^3^ m/s and an upper limit of 2.05 × 10^3^ m/s for the captured orbits (velocity of the GAM at periapsis is 0.246 VU). Orbits with PI lower than 1.0 × 10^3^ m/s resulted in escape trajectories, while values larger than 2.05 × 10^3^ m/s generate collisions (see Figs. 5, 6). For the GAM with 0.336 VU, the captures that occur have PI values larger than 3.2 × 10^3^ m/s and lower than 4.4 × 10^3^ m/s (Figs. 5, 6, right sides). A larger difference is observed in the boundary regions between captured and escaped orbits, because the captured orbits maintain the energy losses due to the constant interaction with the atmosphere, unlike the escape orbits, that interact in a short interval of time with the atmosphere and then leave the proximity of Mars quickly. Figure 5 shows the strong reduction of DV for higher altitudes and lower values of *A/m*, going to zero for the lowest values of *A/m*. This is the first time that an integral index is used to quantify the aerodynamic contribution of the AGAM on Mars, and the results showed that it generates interesting general maps that can be used for mission designers when planning the capacity of the propulsion system or DV budget for the mission. In particular, the PI measures accurately how much the atmosphere reduces the DV for larger altitudes and lowest values of A/m, with the results described by non-homogeneous thin layers. The results of Figs. 5 and 6 were propagated in the prograde direction for 1 day after the approach.

The PI maps for captured orbits are presented in detail in Fig. 6, which shows in more detail the boundary layers of maximum DV before the collision and the minimum DV before the escape region. The layer near the collision zone, for 0.246 VU, indicates a DV around 1.3 × 10^3^ m/s, decreasing in the direction of the escape zone. When the velocity at periapsis is 0.336 VU, the layer has values from 3.4 × 10^3^ m/s to 3.2 × 10^3^ m/s. The color scales in the figures present higher values, which represents a few trajectories that result in this condition after the capture.

**Figure 7:**
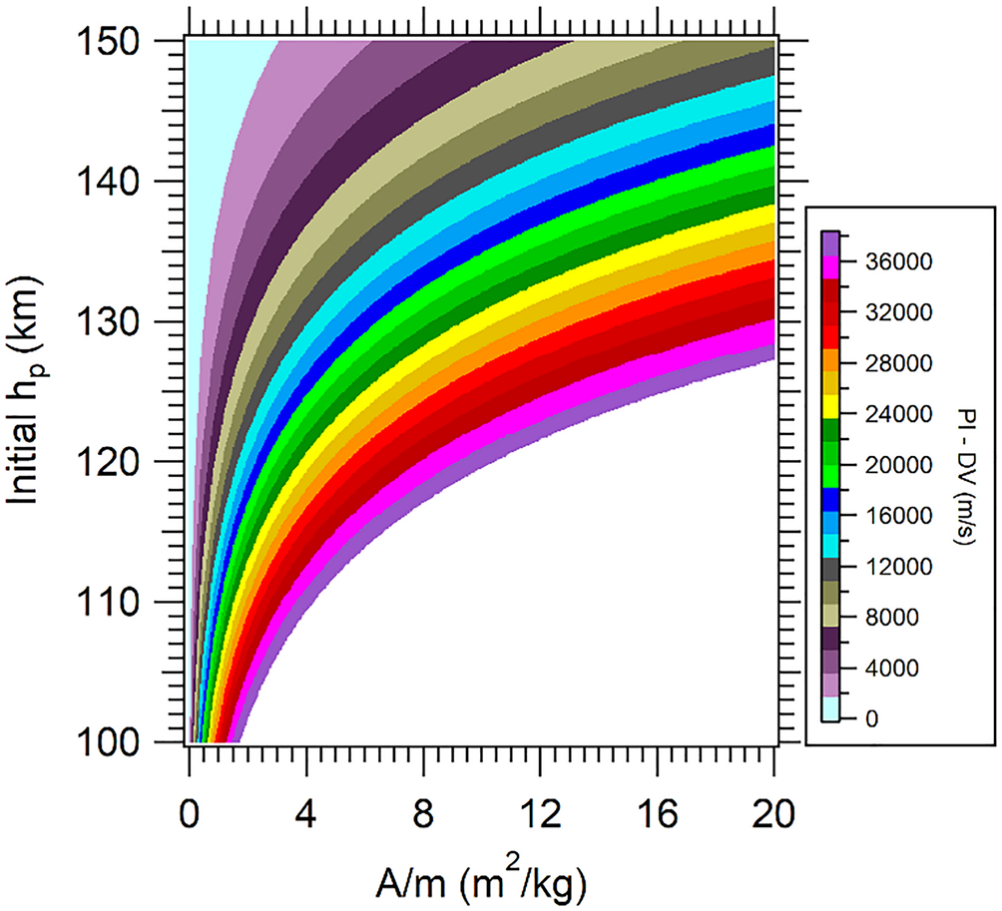
PI maps for VP = 1.89 VU.

In the case of retrograde trajectories, due to the higher values of the velocity (the drag force increases in magnitude with the square of the velocity) therefore, it generates larger values of DV (see Fig. 7). It is also shown that only trajectories with variations between 38.0 × 10^3^ and 37.0 × 10^3^ m/s are captured. A large amount of energy is dissipated due to the interaction with the atmosphere. Figure 7 presents the PI maps for retrograde approaches after 1 day.

To compare the results given by the PI-DV with the single impulse maneuver (without drag), it was determined the eccentricity and semi-major axis of the resulting trajectories for one day after the aerocapture. From the initial trajectory, it is possible to calculate the DV required. For example, in the case of captures with *A/m* = 1.0, at 122.4 km of pericenter altitude and velocity 0.246 VU at periapsis, the semi-major axis resulting from the aerocapture is 6.37819 MR with a PI-DV of 1207.33 m/s. At this point, it is calculated the orbital velocity of the elliptical orbit that the spacecraft would have if the atmosphere were not present. The required DV to transform the orbit, assumed the same final orbit given by the maneuver using the atmosphere, can be calculated from the “vis-viva” equation, and the result is 1115.4 m/s. This is the value saved in the propulsion system by the effect of the atmosphere. Table 2 shows the results of the PI-DV given by drag and the DV required to capture the spacecraft (in GA) and to place it in the same orbit that the atmosphere did. In this case, the values of the impulses are lower than the results obtained using the PI-DV, because the impulses are instantaneous and do not consider the effects of the continuous dissipation from the atmosphere, before the passage by the pericenter.

**Table 2:** DV comparison between a pure impulsive maneuver and AGAM.

A/m (m^2^/kg)	V_p_ (VU)	h_p_ (km)	a (MR)	PI-DV (m/s)	Impulse DV (m/s)
1.0	0.246	122.4	6.37819	1,207.33	1,115.40
11.0		150.0	16.278	1,101.48	992.29
1.0	0.336	112.0	7.41537	3,343.00	3,258.63
11.0		139.0	24.0415	3,240.32	3,138.87

**Figure 8:**
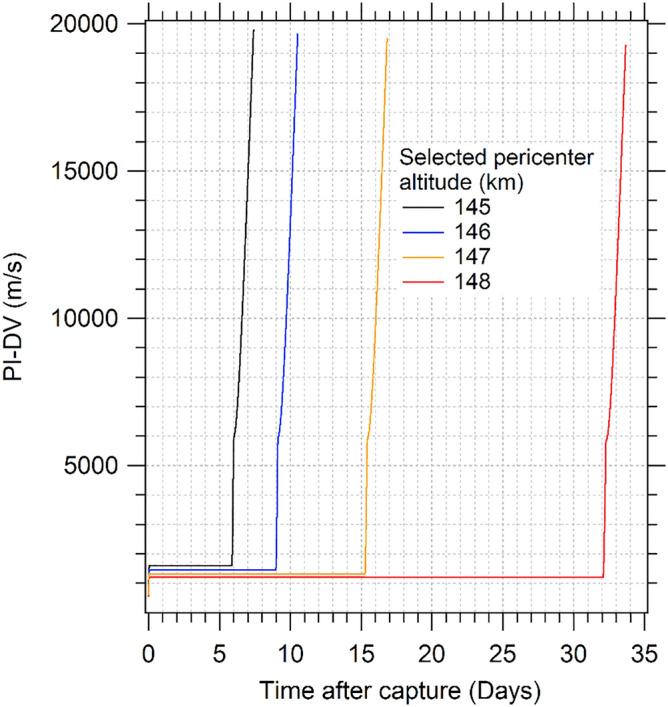
PI-DV as a function of time.

The use of the PI also allows the identification of the variations in the trajectories. In the case of aerocapture, the PI shows an initial large variation in a short time, showing the DV dissipated by the atmosphere during the first approach. In the next days, the variations in DV are negligible compared to the values required for the capture but show small variations that represent the aerobraking. After several days, the largest variation occurs, which represents the change from decay orbit condition to reentry and collision. Then, the PI-DV as a function of time is useful to observe these behaviors, to quantify the DV dissipated by the atmosphere and the duration of the changes. In Fig. 8 it is presented the evolution of the PI-DV for trajectories in prograde direction with 10.0 m^2^/kg and initial velocity at the pericenter of 0.246 VU.

## Conclusions

In this research, for the first time, it was applied the technique known as PI to measure the total impulse obtained from the Martian atmosphere during the close approach. The PI technique allows us to measure the savings that the atmosphere provided during aerocapture, aerobraking, and AGAM. The results show a particularly good agreement between the PI and the savings calculated by the analytical impulsive method. It means that the PI is a valid method to be used to predict the savings generated by drag. This technique should be used to complement the design of future aeromaneuvers.

With the use of the PI-DV technique, it was possible to map the regions of capture, as well as to calculate the DV dissipated by the atmosphere, the savings obtained for the propulsion system, and to quantify the boundary values of the capture regions. Therefore, we can also see the best form to use the atmosphere of Mars for this maneuver and to quantify accurately the savings obtained.

### Ethical approval

The submitted work is original and not have been published elsewhere in any form or language. The work presents the results of a single study. The results are presented clearly, honestly, and without fabrication, falsification, or inappropriate data manipulation. No data, text, or theories by others are presented as if they were the author’s own.

## Data Availability

All data generated or analyzed during this study are included in this published article in form of figures.
